# COVID-19: The impact on urolithiasis treatment in Brazil

**DOI:** 10.1590/S1677-5538.IBJU.2021.0405

**Published:** 2021-09-14

**Authors:** Fernando Korkes, Khalil Smaidi, Matheus Pascotto Salles, Antonio Correa Lopes, Ita Pfeferman Heilberg, Sidney Glina

**Affiliations:** 1 FMABC Faculdade de Medicina do ABC Disciplina de Urologia Santo André SP Brasil Disciplina de Urologia, Faculdade de Medicina do ABC - FMABC, Santo André, SP, Brasil; 2 Universidade Federal de São Paulo Departamento de Nefrologia São Paulo SP Brasil Departamento de Nefrologia, Universidade Federal de São Paulo - UNIFESP, São Paulo, SP, Brasil

**Keywords:** Kidney Calculi, COVID-19, Urolithiasis

## Abstract

**Introduction:**

It has been more than a year since the first case of Covid-19 was diagnosed in Brazil, and its most problematic feature is the oversaturation of the healthcare system capacity. Urolithiasis is a disease that requires timely and appropriate management. The present study aimed to evaluate the impact of the pandemic in hospital admissions for urolithiasis in the Brazilian public healthcare system.

**Materials and Methods:**

In this cross-sectional study, hospital admissions were obtained from the Brazilian Public Health Information system. All hospital admissions associated with urolithiasis diagnosis (ICD-10 N20) between March 2017 and February 2021 were analyzed.

**Results:**

During the COVID-19 outbreak, there was a significant decrease in hospital admissions (p<0.0001). More than 20.000 patients probably did not have the opportunity to undergo their surgeries. The impact of the COVID-19 outbreak on women's admissions was significantly more intense than for men, reducing from 48.91% to 48.36% of the total (p=0.0281). The extremes of age seemed to be more affected, with patients younger than 20 years and older than 60 years having a significant reduction in access to hospital services (p=0.033).

**Conclusions:**

In conclusion, we have noticed a considerable reduction in overall admissions for the treatment of urolithiasis in the Brazilian public healthcare system during the first year of the Covid-19 pandemic. Women and individuals older than 60 years were especially affected. In contrast, we noted a rise in urgent procedures, comparing with the average of the corresponding period of the three previous years. Recovery plans will be needed while returning to activities to handle the impounded surgical volume.

## INTRODUCTION

The covid-19 outbreak has affected nearly every aspect of daily life worldwide, especially in hospitalized patients requiring rehabilitation (1, 2). This acute respiratory syndrome is caused by a newly identified β-coronavirus, the SARS-COV-2, which started in December 2019 in Wuhan, China (3). It has been more than a year since the first case of Covid-19 was diagnosed in Brazil (February 25th, 2020), and that the World Health Organization has declared it a pandemic (March 11th, 2020). As this paper is written, Brazil is considered one of the epicenters of the pandemic, and its most problematic feature is the oversaturation of the healthcare system capacity. Besides the apparent direct effects of the outbreak in healthcare, much has been investigated about its indirect influence over the care of other diseases. Many authors have reported a considerable reduction in the admission rates of non-communicable diseases after the onset of the pandemic (4). However, most of them focused on a short-term crisis (12-16 weeks), whereas Covid-19 has revealed itself more as a long-standing healthcare challenge.

Urolithiasis is a disease that requires timely and appropriate management. It can lead to emergencies that adversely affect kidney function, mainly if associated with infection, potentially leading to death (5). While some authors have found a rise in the rates of complicated ureterolithiasis after the onset of the pandemic (6, 7) or the increase in urgent surgeries for urinary stones, (8) others did not, possibly because of the reduction of presentations to Emergency Departments due to renal colic (9). These observations highlight the patient's fear of being infected as a critical flaw of the COVID-19 pandemic.

In Brazil, it seems that urinary lithiasis has been undertreated during the COVID pandemic, and dramatic consequences are expected within the following years. It is important to quantify this impact, aiming to establish future health politics. The present study aimed to evaluate the adverse effects of the pandemic in hospital admissions due to urolithiasis in the Brazilian public healthcare system.

## MATERIALS AND METHODS

This cross-sectional study reviewed hospital admissions from the Brazilian Public Health Information system (DATASUS). It is a publicly available database that comprises information from the Brazilian Public Health System (SUS). This database includes information from all public health hospitals throughout the country, guaranteeing health support to about 170 million Brazilians.

All hospital admissions associated with urolithiasis diagnosis (ICD-10 N20) between March 2017 and February 2021 were analyzed. Since the COVID-19 outbreak in Brazil was initiated in March 2020, we have considered this month as the beginning of each year, completing one year on the following February. The database was searched for hospital admissions and surgical procedures associated with the treatment with urinary stone disease, under the terms: extracorporeal shock wave lithotripsy (“LITOTRIPSIA EXTRACORPÓREA”), cistolithotripsy (“CISTOLITOTRIPSIA”), lithotripsy (“LITOTRIPSIA”), nephrolithotomy (“NEFROLITOTOMIA”), pyelolithotomy (“PIELOLITOTOMIA”), ureterolithotomy (“URETEROLITOTOMIA”), percutaneous nephrolithotripsy (“NEFROLITOTRIPSIA PERCUTÂNEA”), transureteroscopic ureterolithotripsy(“URETEROLITOTRIPSIA TRANSURETEROSCÓPICA”). Elective and urgent procedures are differentiated in admission statistics. Even though one could expect it as a complication of stone disease, it would not be possible to distinguish those associated with other conditions. Also, patients hospitalized under a different ICD-10 code, such as urinary tract infection or abdominal pain, could not be considered.

As the DATASUS database uses secondary data, ethical approval and informed consent were not required according to resolution 510 of the Brazilian National Health Council. The present study followed the STROBE guidelines for reporting observational studies (10).

Linear regression was used to describe the changes in hospital admissions throughout the years. Statistical analysis was performed using SPSS 13.0 (SPSS for Mac OS X, SPSS, Inc., Chicago, Illinois). Groups were compared with the χ2, T-test and ANOVA. Statistical significance was determined at two-tailed p <0.05.

## RESULTS

**Figure 1:**
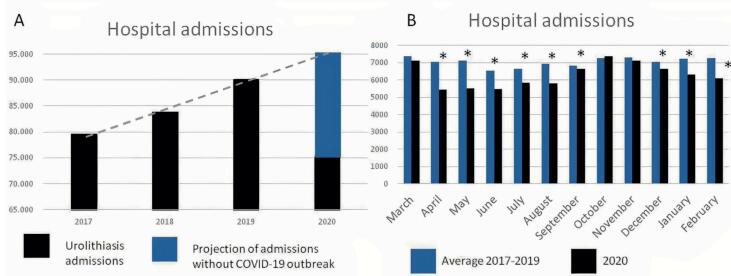
Hospital admissions for urolithiasis from March 2017 to February 2021. A. In blue, projection of admissions growth without the COVID-19 pandemic. B. Monthly comparison between the average number of cases between 2017-2019 and from March 2020 to February 2021.

The number of hospital admissions due to a diagnosis of urinary stone disease has increased during the last years in the SUS system (11). From 2017 to 2019, a mean increment of 6.4% hospital admissions was observed, reaching 90.170 events. These data reinforce previous surveys demonstrating a progressive increase in urolithiasis admissions over the years in Brazil (12). Conversely, during the COVID-19 outbreak, there was a significant decrease in hospital admissions with urolithiasis diagnosis compared to preceding years (p <0.0001, Figure-1). From March 2020 to February 2021, there have been 75.461 admissions. More than 14.000 patients probably did not have the opportunity to undergo their surgeries if we consider 2019's numbers (and more than 20.000 for a net growth of 6.0%).

**Figure 2:**
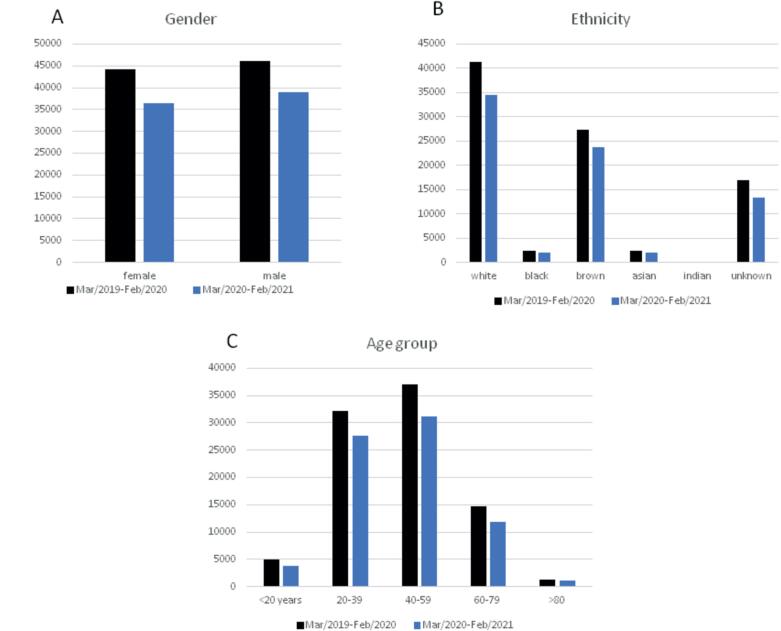
Effect of the COVID-19 outbreak in hospital admissions for urolithiasis according to gender (A), ethnicity (B), and age (C).

Monthly comparisons demonstrated that case reduction occurred throughout the year after the onset of the COVID-19 outbreak, except during October and November (Figure-2).

The impact of the COVID-19 outbreak on women's admissions was significantly more intense than for men. While the 36.496 female admissions represented 48.36% (a reduction of 1.0% compared to previous years), male admissions increased 1.0% instead during the pandemic. Women's admissions reduced from 48.91% to 48.36% of the total (p=0.0281, Table-1).

**Table 1:** Hospital admissions for urolithiasis according to age groups, gender and ethnicity (Brazil, 2019 vs. 2020)

		2019		2020			
Age group		N	%	N	%	χ²	p value
	< 20 years	4924	5.46%	3719	**4.93%**	23.44	**<0.0001**
	20-39	32223	**35.74%**	27700	36.71%	16.76	**<0.0001**
	40-59	37028	41.06%	31197	41.34%	1.29	0.256
	> 60	15995	17.74%	12845	**17.02%**	14.62	0.0001
**Gender**							
	Female	44099	48.91%	36496	**48.36%**		
	Male	46071	51.09%	38965	51.64%	4.82	0.0281
**Ethnicity**							
	White	41286	45.79%	34581	45.83%	0.02	0.8875
	Black	2293	2.54%	1953	2.59%	0.32	0.5716
	Brown	27249	**30.22%**	23655	31.35%	24.49	**<0.0001**
	Asian	2438	2.70%	1924	2.55%	3.75	0.0528
	Indian	68	0.08%	57	0.08%	0.01	0.9203
	Unknown	16836	18.67%	13291	**17.61%**	30.98	**<0.0001**

The effect of the pandemic on hospital admissions was also unequal according to age groups. The extremes of age seemed to be more affected. Patients younger than 20 years and older than 60 years significantly reduced hospital services access than patients aged 20-59 years (Table-1).

Brazil is very rich in miscegenation, and individual race relies on self-report. Hereupon, statistics reveal more patients without proper self-report definition during the pandemic.

Elective surgical procedures undertaken during 2020 have been significantly reduced compared to the average yearly operations during 2017-2019 (9.720 vs. 14.290, p <0001). On the other hand, urgent procedures have significantly increased when comparing 2020 vs. 2017-2019 (9.379 vs. 8.947, p=033).

**Figure 3:**
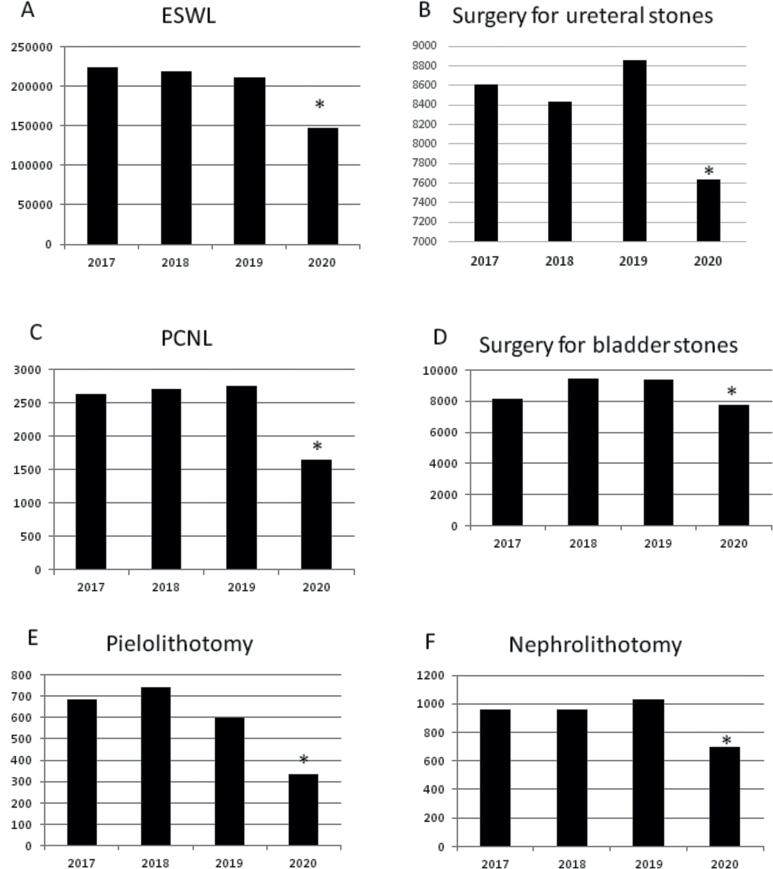
Frequency of surgical procedures performed (March-February, 2017-2021). The procedures with respectives p-value: Surgeries to remove ureteral (p<.0001), or bladder stones (p<.0001), percutaneous nephrolithotomy (PCNL, p=.043), nephrolithotomy (p<.0001), pyelolithotomy (p<.0001), andextracorporeal shock wave lithotripsy (ESWL) (p<.0001).

All surgical procedures performed to treat urinary stone disease were significantly less performed during 2020 (Figure-3), despite a historical trend of increasing procedure numbers (13). This includes surgeries to remove ureteral (p <0001), or bladder stones (p <0001), percutaneous nephrolithotomy (PCNL, p=043), nephrolithotomy (p <0001), pyelolithotomy (p <0001), and extracorporeal shock wave lithotripsy (ESWL) (p <0001).

## DISCUSSION

Brazil faced a COVID-19 outbreak with two significant waves and a collapse in the health system throughout the country. Even though there has been an effort to maintain specific treatments, such as oncologic and emergency conditions, many diseases were left untreated. The main findings of the present cross-sectional study were a reduction of 16.3% in hospital admissions for urolithiasis treatment, especially among women when compared to men (women admissions reduced from 48.91% to 48.36%) and a shift to a 5% higher need of urgent procedures compared to the preceding year.

The significant reduction in hospital admissions due to urinary stone disease in Brazil during the first year of the COVID-19 outbreak indicates that more than 20.000 Brazilians could not have their stones treated during this first year of the pandemic. The months of October and November were the least affected, probably due to the decline in pandemic burden as infection rates gradually decreased before they started to rise again - and healthcare facilities could resume their activities for a brief period. Many factors influenced the heterogeneous response to the pandemic all over the continental dimensions of the country. The diversity of the resources and the containment measures taken have affected its burden differently in each region of Brazil. A recent study before the COVID-19 era (14) had shown that patients living in low human development index (HDI) areas are more prone to develop struvite stones, possibly due to more inadequate access to healthcare. Nevertheless, a reduction in admissions for urolithiasis depicted in the current series was persistently observed, and all modalities of surgical procedures have been affected.

Of note, women seemed to be even more affected than men in their urinary stone treatments (p=0.0281). Women's admissions reduced from 48.91% to 48.36% of the total (p=0.0281). Other investigators did not report such findings (6, 7). Although the reasons for our results are still unclear, one can speculate that the pandemic might have hit harder in women than men. The parts of the economy that suffered the most were the ones women predominate.

Other groups that were also affected were the extremes of ages. Individuals younger than 20 years and older than 60 years significantly reduced access to hospital services compared to patients with 20-59 years. Concerning the elderly, they were more vulnerable and isolated during the last year and probably might have taken less care of their health as a whole.

Extensive studies have reported that hospitals in many countries have suspended or deferred all elective surgeries to maintain critical care capacity (15–18). It was estimated that more than 28 million procedures were canceled or postponed worldwide due to Covid-19 in its three worst months of 2020 (19). This study also observed a considerable reduction in elective admissions for urolithiasis in Brazil when comparing 2020 and the average of the preceding three years (9.720 vs. 14.290, respectively, p <0001). In the context of an oversaturated and heterogeneous public healthcare system, as Brazil's example, it reflects the need for better allocation of the already limited resources (20). Hospital beds and entire wards were redesigned, and material and human resources were reallocated, leading to the underdiagnosis of many conditions by postponing non-urgent cases (21). Many clinics were closed or have seen a significant reduction in their volume of outpatient care, increasing the difficulty in accessing the system (22).

Reduction in admission rates for all causes has been reported. Physical and social distancing (4, 6, 23) and fear of contamination (24, 25) have led people to avoid medical care. This overall reduction might be due to less hospitalization of mild to moderate cases (21), lower demand by patients, or the medical attempt to relieve the system (5). The latter might even be a leading reason for reducing hospitalization by urolithiasis in this period (6). These same reasons could explain the relative rise we observed in urgent procedure rates since more complex cases might have arisen from patients with delayed diagnosis and suboptimal treatments (16). The 5% increment in urgent procedures to treat urolithiasis during 2020 (p=033) might become even higher during the months or years to come since there might be a massive volume of accumulated cases.

In summary, as the Impact of Covid-19 escalates, the progressive postponement of elective procedures is widely adopted. The term “elective” is often vague and open to interpretation. Some “elective” procedures must not be deferred for long time at the expense of a significant increase in morbidity and mortality. For many interventions, the line between urgent and elective can only be traced retrospectively (24). It will continue to challenge urology and nephrology departments to manage cases that lie somewhere between these two categories (26). As an attempt to fill this gap, there have been attempts to group tier-based priorities better to guide the time of intervention in a way to minimize patient risk (5, 27–29), including recommendations from the European Association of Urology dividing patients into categories of either emergency, high, intermediate or low priority (30). They aim to prioritize patient safety, weighing the benefits and risks of postponing diagnosis and treatment with Covid-19 exposure (31). It is difficult to develop recommendations that fit all centers due to each one's particularity regarding the number of patients, the number of surgical staff and anesthesiologists, availability of hospital beds, operating rooms, mechanical ventilators, and medication supplies, as well as the severity and burden of Covid-19 in each region at any given period (5, 31). Most recommendations do not recon a more prolonged crisis.

The definitive stone treatment is still a matter of debate during the Covid-19 pandemic. Some urologists prefer active stone treatment over temporary drainage to reduce the number of emergency room visits, except if an infection is present or staged treatment is expected (5, 7). Others prefer to defer all procedures to treat urinary stones until the end of the Covid-19 pandemic, with temporary drainage only if indicated (8, 17). If aerosolization and risk of viral transmission is a concern, endoscopic procedures are less hazardous than open or laparoscopic approaches (32). A fair surgical judgment may help reduce the load on healthcare systems and optimize the care towards fighting this crisis.

There should be initiatives towards health education for the general population concerning preventing Covid-19 and managing all other health care, especially in reducing collateral damage caused by delayed urgent diagnosis due to fear. This emergency public health crisis could be recognized as an opportunity to develop and better integrate telemedicine care such as telemonitoring as a standard in healthcare (33), as almost 40% of Brazilian urologists have experienced (34, 35). It also may be a method to boost efficiency and guide the prioritization of surgical lines (26). As a long-standing healthcare challenge, all urologists should be confronted with the need to organize the schedule of a long waiting list of patients (16, 36) and manage more complicated cases (5, 26).

Our study has some limitations. The inability to check data accuracy and the lack of clinical data of the patients limits further analysis. Additionally, the cross-section retrospective design does not allow us to follow these patients. Moreover, present data is restricted to hospitalization. According to Kachroo et al. (37), based on presentations to emergency departments in the US, there has been an even higher reduction (36%) in urinary stone disease cases compared to a pre-COVID era. However, the strength of the present study relies on the high number of patients analyzed. Moreover, it brings important information to help understand the effects of the COVID-19 outbreak in patients with urolithiasis and might be valuable in predicting the best health politics in the years to come. The present study can help us understand the outbreak's impact on patients with urinary stone disease and possibly help predict what is about to come during the ensuing years.

In conclusion, we have noticed a considerable reduction in overall admissions for the treatment of urolithiasis in the Brazilian public healthcare system during the first year of the Covid-19 pandemic, mainly due to suspension and deferral of elective surgeries. In contrast, we noted a rise in urgent procedures, comparing with the average of the corresponding period of the three previous years. Women and individuals older than 60 years were especially affected. Recovery plans will be needed while returning to activities to handle the impounded surgical volume.
